# Innovation hubs and young adults’ employment in fragile and conflict-affected Somalia: evidence from Mogadishu

**DOI:** 10.3389/fsoc.2026.1842865

**Published:** 2026-07-16

**Authors:** Adnan Abdukadir Ahmed, Mohamed Liban Isak

**Affiliations:** Faculty of Management Sciences, SIMAD University, Mogadishu, Somalia

**Keywords:** conflict-affected contexts, digital skills, entrepreneurship support, fragile states, freelancing programs, innovation hubs, skills development, Somalia

## Abstract

**Purpose:**

Youth unemployment remains one of the most pressing socioeconomic challenges in fragile and conflict-affected contexts such as Somalia, where prolonged instability, weak institutions, and limited labor market opportunities continue to constrain young adults’ access to sustainable employment. In response to these challenges, innovation hubs have emerged as important platforms for skills development, entrepreneurship support, and freelancing opportunities. However, empirical evidence on the relationship between innovation hub initiatives and employment outcomes remains limited. This study examines the association between innovation hub services, specifically skills development and training, entrepreneurship support, and freelancing programs, and young adults’ employment outcomes in Mogadishu, Somalia.

**Methodology:**

A quantitative cross-sectional research design was employed using primary data collected through a structured online questionnaire. The study focused on young adults who had participated in innovation hub programs, with a sample of 360 respondents selected through purposive sampling. Data were analyzed using SPSS through descriptive statistics, reliability analysis, Pearson correlation, and multiple linear regression to examine the relationships among the study variables.

**Findings:**

The findings reveal that all three innovation hub services are positively and significantly associated with young adults’ employment outcomes. Skills development and training emerged as the strongest predictor, followed by freelancing programs and entrepreneurship support. The results further suggest that participants reported improved employment experiences after engaging in innovation hub programs. The regression model demonstrated a moderate level of explanatory power, indicating that the three predictors jointly account for a meaningful proportion of variation in employment outcomes.

**Implications:**

The study provides important insights for policymakers, educators, and practitioners by highlighting the potential role of innovation hubs in addressing youth unemployment in fragile and conflict-affected settings. Strengthening practical skills training, expanding freelancing opportunities, and supporting entrepreneurship development may enhance employment opportunities for young adults. The findings further support the integration of innovation hubs into broader employment and workforce development strategies in Somalia.

## Introduction

Young adults constitute a vital segment of any nation, acting as key drivers of social and economic progress. They typically exhibit high levels of creativity, innovation, adaptability, and productivity, and they represent the upcoming generation of professionals, entrepreneurs, and leaders. According to the World Bank (2022) an estimated 1.8 billion individuals, roughly 23% of the world’s 8 billion people, are between 15 and 29 years old. Adekunle et al. (2023) emphasize that weak economic systems, entrenched inequalities, and inadequate educational structures significantly restrict employment opportunities. A major contributor to youth unemployment in Sub-Saharan Africa is the mismatch between the skills provided by educational institutions and the competencies required by employers (Grace et al., 2017).

Previous studies suggest that entrepreneurship support, digital skills development, and practical training initiatives can play an important role in improving youth employment opportunities, particularly in developing contexts (Alawad et al., 2020; Bignotti and Le Roux, 2018; Dinika, 2024). However, the rapid increase in university graduates without corresponding job creation continues to intensify graduate unemployment challenges across many African countries (Brown et al., 2010). Youth unemployment is one of Somalia’s most urgent development issues. The country’s prolonged civil conflict, unstable political environment, and disrupted economy have severely weakened its labor market and hindered socioeconomic progress. Graduate unemployment, in particular, has become a significant societal concern, with potential links to increased crime and persistent social tensions (Gelle et al., 2021). A significant number of Somali graduates experience prolonged periods of joblessness. According to Hassan (2022) reported over two-thirds of unemployed individuals had been without work for a minimum of eight months, and more than half were actively seeking employment throughout that duration.

Many small businesses prefer informal recruitment methods, relying on family networks, close acquaintances, and clan leaders, rather than publicly advertising vacancies. Over 60% of youth express intentions to migrate to seek better employment prospects. Cultural and clan-based biases, along with insufficient practical training opportunities, further intensify joblessness in the country (The Ministry of Planning, Investment, and Economic Development, 2020). Unemployment remains one of the country’s most pressing socioeconomic issues and contributes to numerous social problems (Salad, 2023). Heritage Institute for Policy Studies (2022) identifies a significant skills mismatch between the competencies required by productive sectors and those possessed by Somali graduates, creating a major barrier to youth employment. Unemployment is primarily driven by factors such as limited job creation, mismatches between available skills and industry needs, unstable economic environments, and inadequate work experience (Ahmed, 2024). Heritage Institute for Policy Studies Studies (2022) highlights that in Somalia, the gap between the competencies required by employers and the qualifications possessed by young adults significantly restricts youth employment, as many graduates are not adequately prepared for the demands of the labor market.

Somalia continues to face high levels of youth unemployment due to prolonged conflict, political fragility, and limited formal employment opportunities. In response to these challenges, innovation hubs have emerged as practical platforms for equipping young adults with market-oriented skills, entrepreneurship support, and access to freelancing opportunities.

In response to these challenges, innovation hubs such as SIMAD iLab and iRise Hub have emerged as important platforms for providing digital skills training, entrepreneurship support, and freelancing opportunities for young adults in Somalia. Despite the growing prominence of these hubs, limited empirical research has examined how their programs are associated with employment outcomes among young adults in the Somali context. A more detailed discussion of the theoretical and empirical literature related to youth unemployment, innovation hubs, entrepreneurship support, and freelancing programs is presented in the following literature review section.

This research addresses the following questions (RQs):

RQ1: To what extent are skills development and training programs associated with employment outcomes for young adults in Somalia?
RQ2: How is entrepreneurship support provided by innovation hubs associated with young adults’ employment?
RQ3: How are freelancing programs associated with employment prospects among young adults in Somalia?

## Literature review

### Educational and skills-related determinants of youth unemployment

Youth unemployment continues to pose a major global challenge, largely driven by a persistent misalignment between the skills offered by educational institutions and what is demanded in the labor market. Many young people complete formal education without acquiring the practical, technical, or entrepreneurial competencies required for available employment opportunities. This disconnect often results in prolonged unemployment or underemployment among youth. To facilitate a smoother transition from education to work, it is essential that academic institutions align their curricula with current labor market requirements (Murat and Şahin, 2011). One of the most influential drivers of youth unemployment is the gap between educational outcomes and employer expectations. Günaydın and Çetin (2015) argue that this mismatch creates a supply-side imbalance in labor markets, where graduates possess qualifications that do not meet industry needs. Similarly, Gebretsadik (2016) identifies skills mismatch, inadequate entrepreneurship education, and limited employment opportunities as key contributors to youth unemployment in emerging economies. Okolocha et al. (2020) noted that rising unemployment among youth is often linked to insufficient complementary skills acquired through formal education. Likewise, Alawad et al. (2020) find that in Jordan, the growing rate of youth unemployment is largely attributed to the disparity between skills gained through education and those required by employers. Ismail (2011) further asserts that graduate quality is a critical factor in the unemployment crisis, noting that strong academic performance alone is no longer sufficient to secure employment in today’s labor markets.

### Innovation hubs and post-conflict reconstruction in Somalia

Across the globe, innovation hubs have emerged as important platforms for developing and implementing creative solutions to address both societal challenges and market needs (Cherunya and Ahlborg, 2020). Scholars have conceptualized innovation hubs from multiple perspectives. The earliest documented use of the term *“innovation hub”* can be traced back to 2001, with the establishment of The Innovation Hub in South Africa (Baark and Sharif, 2006). Following the collapse of the Somali state in 1991 and the outbreak of prolonged civil conflict, the country experienced a severe breakdown of formal educational systems and institutional structures. Academic institutions, vocational training centers, and university resources were among the most affected sectors due to widespread infrastructural destruction (Abdi, 1998). As Somalia continues its gradual recovery and development, efforts to rebuild human capital have become increasingly important. In recent years, innovation hubs have emerged as key initiatives aimed at addressing youth unemployment by equipping young Somalis with relevant skills, practical training, and pathways to employment. Established in 2017, iRise Hub is the first community-based innovation hub in Mogadishu. Its mission is to harness Somalia’s human capital and stimulate economic transformation by promoting innovation and entrepreneurship. The hub focuses primarily on the digital economy, coding, education, and capacity-building programs. Through its emphasis on resilience and innovation, iRise Hub has become an integral component of the local entrepreneurial ecosystem and remains committed to supporting Somali youth throughout various stages of their personal and professional development. SIMAD iLab is Somalia’s first university-affiliated innovation hub, established in 2019 under the ownership and management of SIMAD University, one of the country’s leading higher education institutions. The hub provides a range of services, including professional skills development programs, startup incubation, and business acceleration initiatives, primarily serving young innovators and entrepreneurs in Mogadishu.

Existing literature generally suggests that innovation hubs, entrepreneurship support initiatives, digital skills development programs, and online freelancing opportunities may contribute to improving youth employment outcomes, particularly within developing economies characterized by high unemployment and limited formal labor-market opportunities (Alawad et al., 2020; Bignotti and Le Roux, 2018). However, much of the existing evidence has primarily focused on relatively stable economic environments, with limited empirical attention devoted to fragile and conflict-affected contexts such as Somalia, where political instability, weak institutional structures, and constrained labor markets create significantly different employment conditions for young adults. Furthermore, although prior studies frequently emphasize the positive potential of digital entrepreneurship and gig-based work in expanding access to employment opportunities, growing scholarly debate also highlights concerns relating to labor precarity, unstable income, inconsistent work opportunities, algorithmic dependency, and limited social protection within platform-based labor markets (Wood et al., 2019; Sutherland and Jarrahi, 2017; Graham et al., 2017). In many developing contexts, online freelancing may provide important income-generating pathways for youth while simultaneously exposing workers to intense global competition, irregular earnings, and uncertain long-term economic security. Despite these emerging debates, limited research has critically examined how innovation hub services interact with employment-related outcomes within fragile labor-market environments such as Mogadishu, Somalia. Therefore, the present study contributes to the literature by empirically examining the associations between skills development and training, entrepreneurship support, freelancing programs, and young adults’ employment-related outcomes within a fragile and conflict-affected context.

### Theoretical framework

#### Human capital theory

Within the labor market, individuals differ in the levels of education, experience, skills, competencies, and career aspirations they bring to employment. Bontis et al. (1999) described human capital as the collective human attributes within an organization, encompassing knowledge, expertise, and intellectual abilities that shape its distinctive value. Similarly, McConnell et al. (2009) argue that individuals with higher levels of education and training are capable of generating greater productive output than those with limited qualifications. Human capital theory is widely recognized for its contribution to improving organizational performance, as institutions rely heavily on the knowledge, skills, and capabilities of their workforce as key sources of value creation. The intellectual foundations of human capital theory can be traced back to the eighteenth century, when Smith (1776) first emphasized the economic importance of skills and productive labor in industrial development. The concept was later formalized by Schultz (1961), who introduced the idea of “investment in human capital” in the *American Economic Review*. Human capital theory gained further prominence following Gary. Becker’s seminal contributions, which earned him a Nobel Prize and solidified the theoretical framework linking education and training to productivity and earnings. Becker argued that differences in education and skill acquisition result in varying levels of individual contribution and income. As individuals accumulate knowledge, skills, and competencies, their prospects of securing high-quality employment increase (Blair, 2011). Individuals invest in their own education and skill acquisition with the expectation that these investments will improve their employability, career advancement, and income potential.

#### Theory of effectuation

The theory of effectuation is a framework of entrepreneurial decision-making developed by Sarasvathy (2001). Effectuation describes the logic by which entrepreneurs create new ventures under conditions of deep uncertainty, situations where the future is not merely risky in a probabilistic sense but genuinely unknowable (Sarasvathy, 2001; Sarasvathy, 2008). Effectuation inverts this logic: the entrepreneur starts with available means—who they are, what they know, and whom they know—and allows goals to emerge iteratively through action and stakeholder engagement (Sarasvathy, 2001; Sarasvathy, 2008). The theory is organized around five principles: (1) *Bird-in-Hand*: begin with existing means rather than waiting for ideal resources; (2) *Affordable Loss*: commit only what one can afford to lose, rather than maximizing expected returns; (3) *Crazy Quilt*: build partnerships with self-selecting stakeholders who co-create the venture; (4) *Lemonade*: leverage unexpected contingencies as opportunities rather than threats; and (5) *Pilot-in-the-Plane*: focus on shaping the controllable aspects of an unpredictable future (Sarasvathy, 2008; Read et al., 2009).

Effectuation theory is particularly suited to explaining entrepreneurial behavior in resource-constrained environments such as those found in developing countries (Ali et al., 2021; Dawa and Marks, 2024). In such settings, entrepreneurs face non-conducive business environments, scarce finance, limited technological infrastructure, and weak institutional support, conditions that render causation-based planning unreliable (Ali et al., 2021). As Ali et al. (2021) argue, “enhancing entrepreneurial effectuation can enable the entrepreneurs of developing countries to successfully create such businesses that are not only efficient in resource utilization but also have a competitive outlook.” Effectuation provides an alternative framework that starts from the youth’s existing reality. As USAID/YouthPower Learning (2020) documented, effectuation-based entrepreneurship education and training programs help young people in low- and middle-income countries to begin with available resources, build partnerships iteratively, and manage risk through affordable loss rather than expected-return calculations. Owusu and Janssen (2013) studying social entrepreneurship in West Africa, found that effectuation and bricolage approaches enabled entrepreneurs to overcome resource constraints in uncertain environments through exploration, experimentation, and flexibility.

In the Somali context, where institutional voids, political instability, and limited formal employment structures characterize the entrepreneurial landscape, the effectual logic is especially relevant. Young adults in Mogadishu typically lack the capital, market data, and institutional support required for causation-based planning. Instead, they must rely on personal networks, available skills, and iterative experimentation, precisely the means-driven approach that effectuation describes.

#### Gig economy theory

The gig economy has emerged as a central subject in labor market research and policy debate over the last decade. It encompasses digitally enabled platforms that connect service providers with clients for temporary, project-specific engagements. Work in this economy is defined by short-term, task-oriented assignments arranged through digital platforms, rather than conventional long-term employment contracts (Donovan et al., 2016). The term “gig” itself refers to a piece of work carried out within a specified timeframe or for a particular task (Mehta, 2023). For many young people, especially those in settings with limited economic resources, gig work represents a comparatively low-barrier entry point into income-earning activity (Pesole et al., 2018). Platforms such as Upwork and PeoplePerHour allow individuals and businesses to hire remote workers for short-duration projects spanning diverse skill domains (Blaising et al., 2021). Newcomers to online freelancing, often called “newbies” or “noobs,” typically begin by exploring it as a secondary income stream, treating it as part-time work alongside locally available employment options (Sison and Lavilles, 2018).

Scholars increasingly acknowledge the gig economy’s role in fostering youth employment and skills acquisition by providing flexible avenues for earning income and experimenting with entrepreneurship (Ghafur et al., 2024). It serves as a practical channel for absorbing unemployed young people who are willing to work but encounter structural obstacles in accessing formal labor markets. The growth of gig-based employment holds considerable potential to improve aggregate employment figures at the national level (Mehta, 2023). According to Manyika et al. (2015) estimate that digital labor platforms have the potential to create job opportunities for tens of millions of people worldwide in the coming decades. Gig economy theory suggests that digital platforms and flexible, short-term work models provide feasible alternative employment pathways for young people, especially in low-income and fragile contexts like Somalia. In line with this, innovation hubs across Somalia have launched freelancing programs, digital skills training initiatives, and support mechanisms for accessing global online labor markets, thereby fueling gig economy growth.

#### Hypothesis development

Skills development plays a decisive role in shaping employment opportunities for young people. In an increasingly competitive labor market, the acquisition of relevant and up-to-date skills is critical for securing meaningful employment. Rapid business expansion and continuous technological change require young job seekers to regularly upgrade their competencies in order to remain employable. Kumar (2022) emphasizes that skills development is closely linked to employability outcomes. In this study, skills development and training programs are defined as structured learning initiatives provided by innovation hubs that aim to enhance young adults’ employability by equipping them with practical, market-oriented competencies. Betcherman and Khan (2015) identify skills enhancement as a central component of youth employment strategies across Sub-Saharan Africa. Empirical studies consistently demonstrate the positive impact of skills training on youth employability. Sridevi and Kumar (2024) report that skills development programs significantly improve individuals’ chances of employment by providing essential job-related competencies. Lalitha (2019) similarly finds that skill training enhances employment prospects and contributes to improved living standards among young people. Medun and Bello (2024) report a strong positive relationship between skills acquisition and youth employability (r = 0.788, *p* = 0.05), concluding that skills development substantially enhances employable capacities and supports job creation. These findings reinforce the effectiveness of skills acquisition as a strategy for addressing youth unemployment.

Scholars further emphasize the importance of education and training in enabling young people to secure decent employment in formal labor markets. Okada (2012) argues that continuous education and skills enhancement are essential for accessing stable employment opportunities. Enu-Kwesi (2013) similarly stresses the need for integrated educational and training initiatives that develop the competencies, knowledge, and attitudes required for productive employment. In the digital era, employability standards continue to rise, with increasingly advanced skill sets becoming prerequisites for labor market participation (Yigitcanlar, 2008). Zaman (2015) highlights the importance of aligning skills development with vocational education, noting that improved skills significantly enhance young people’s access to employment opportunities. Betcherman and Khan (2015) reaffirm that skills development initiatives remain critical for improving youth employment outcomes across Sub-Saharan Africa.

*H1*: Skills development and training programs are positively associated with young adults’ employment in Somalia.

Entrepreneurship support has gained growing recognition as a vital approach to tackling youth unemployment globally. Policymakers and development organizations recognize that formal employment markets frequently lack the capacity to absorb all graduates, which has led to the creation of programs that encourage young people to explore entrepreneurial career paths. Okoro (2020) argues that entrepreneurship education enables youth to unlock their potential while simultaneously contributing to solutions for unemployment-related societal challenges. Empirical findings from Okoro et al. (2022) indicate that graduates exposed to entrepreneurship training are three times as likely to establish new businesses compared to those who have not received such training. Entrepreneurs not only generate self-employment opportunities but also create jobs for others, thereby playing a broader role in reducing unemployment. Consequently, youth entrepreneurship is increasingly viewed both as a pathway to individual employment and as a key driver of job creation within the economy (Yershova et al., 2022). Ndungu and Anyieni (2019) further observe that countries experiencing growth in entrepreneurial competencies tend to exhibit lower unemployment rates, highlighting entrepreneurship as a sustainable, long-term response to youth unemployment. Shefiu (2016) emphasizes that well-structured entrepreneurship programs can substantially enhance self-employment and job creation among young people.

In a related study, Gielnik et al. (2020) demonstrated that entrepreneurship education strengthens entrepreneurial intention, boosts self-confidence, and improves the ability to identify opportunities, concluding that thoughtfully structured programs can lower youth unemployment by stimulating business formation. Akoto (2023) supports this view, emphasizing that engaging young people in real ventures and simulated business activities significantly enhances their confidence and entrepreneurial capabilities. Malenya et al. (2025) further report that startup incubators play a crucial role in enabling youth entrepreneurship by offering mentorship, access to finance, and business support services, thereby directly contributing to job creation. In the African context, entrepreneurship is widely regarded as a key engine for employment generation and economic growth. According to Chidiebere et al. (2014) entrepreneurship initiatives have the potential to offer supportive ecosystems and the structural foundations that empower youth to establish enterprises and alleviate unemployment continent-wide. Kluve et al. (2017) similarly notes that training in business skills is a core component of many youth employment interventions, as it equips young people with the competencies required to initiate, manage, and scale entrepreneurial ventures. Collectively, this body of literature underscores the importance of entrepreneurship support services in enhancing employment outcomes for young adults.

*H2*: Entrepreneurship support services provided by innovation hubs are positively associated with young adults’ employment.

Digital freelancing marketplaces like Fiverr and Upwork have opened up flexible earning opportunities for young people, although they simultaneously raise concerns regarding job stability and employment protection. Freelancers often operate without long-term contracts, exposing them to income instability and intense global competition, where services may be underpriced and legal protections are limited (Esakkiammal et al., 2024). Donina et al. (2022) describe freelancing as a widely adopted form of self-employment that functions not only as a temporary income source but also as a sustainable career pathway (Akhmetshin et al., 2018). Young adults now constitute a significant share of the freelance workforce, with individuals aged 18 to 30 accounting for approximately 58% of freelancers worldwide (Kadnichanskaya and Galkina, 2019). Freelancing has enabled many young graduates to generate income, acquire work experience, and develop skills through flexible and self-directed work arrangements (Riaz et al., 2021).

Perampalam et al. (2017) suggest that digital labor platforms have the potential to transition unemployed young people into full-time freelance work. In developing economies, online freelancing has emerged as a powerful mechanism for job creation and income generation, significantly improving livelihoods (Perampalam et al., 2017). Several studies emphasize that freelancing offers a broad range of opportunities for young people, particularly when they are equipped with relevant digital and professional skills (Wood et al., 2019; Cieslik et al., 2022; Tay and Mohamad, 2022; Anwar and Graham, 2021). Providing youth with freelancing-related competencies enhances their employability and promotes self-reliance. In Sub-Saharan Africa, the gig economy is expanding rapidly, presenting favorable conditions for reducing unemployment and underemployment among young adults (Ramalingam, 2016). Swathis (2023) further argues that many unemployed youth can access meaningful economic opportunities through participation in the gig economy.

In this study, freelancing programs refer to structured initiatives offered by innovation hubs that equip young adults with digital and professional skills required to access online labor markets and perform project-based work through global freelancing platforms. These programs typically include training in digital skills such as content creation and digital marketing, as well as practical guidance on profile creation, portfolio development, client communication, bidding strategies, and managing freelance projects on platforms such as Upwork and Fiverr. Through these components, freelancing programs aim to enhance self-employment opportunities and improve young adults’ access to flexible income-generating work.

Beyond employment creation, freelancing provides legitimate, flexible, and legally recognized income opportunities for young people (Porwal and Kumar, 2023). Prasad (2016) identifies freelance work as one of the most viable employment options for graduates who struggle to secure traditional jobs. Balaram et al. (2017) similarly observe that gig-based work serves as an entry point into the labor market for young individuals, facilitating their transition into the workforce.

*H3*: Freelancing programs offered by innovation hubs are positively associated with young adults’ employment.

#### Conceptual framework

**Figure 1:**
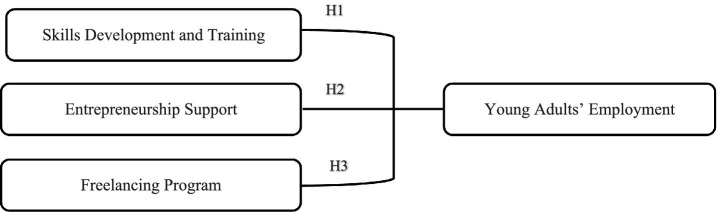
Conceptual framework.

Based on the reviewed literature and theoretical foundations, the authors developed the conceptual framework presented in Figure 1. The model illustrates the relationships between the core services delivered by innovation hubs, and their collective influence on young adults’ employment outcomes.

## Methodology

### Study design and measurement

This study adopted a quantitative research design to examine the influence of innovation hubs on the employment outcomes of young adults in Somalia. A quantitative approach was considered suitable as it enables hypothesis testing and the empirical assessment of relationships among variables. Primary data were collected directly from participants to ensure relevance and accuracy. Data collection was conducted using a structured online questionnaire administered through Google Forms, a widely accepted tool in quantitative research for systematic data gathering. The questionnaire was distributed to respondents through the participating innovation hubs. Ethical approval for this study was obtained from the Research Ethics Committee of SIMAD University. The study was conducted in accordance with internationally accepted ethical standards for research involving human participants. All participants were informed about the purpose of the study and provided voluntary informed consent. Participants were assured of the confidentiality and anonymity of their responses. All data were reported in aggregate form to prevent the identification of individual respondents. The data collection period spanned from August 2024 to March 2025.

In this study, the term “young adults” was used to refer to individuals aged between 20 and 35 years who participated in innovation hub programs in Mogadishu, Somalia. Although the broader literature and policy discussions frequently use the term “youth” when addressing employment challenges among younger populations, the present study uses the term “young adults” to more accurately reflect the age composition of the study participants and maintain terminological consistency throughout the manuscript.

### Sample and data collection

The study employed purposive sampling, a non-probability sampling technique, to deliberately select participants who were directly involved in innovation hub programs. Participants were selected using purposive sampling because the study specifically targeted young adults who had direct experience participating in innovation hub programs offered by SIMAD iLab and iRise Hub in Mogadishu, Somalia. To be included in the study, respondents were required to have previously participated in at least one innovation hub program related to skills development, entrepreneurship support, or freelancing initiatives. Individuals without prior participation in innovation hub activities were excluded from the study. The questionnaire link was distributed online through the support of the participating innovation hubs and shared with eligible participants through institutional communication channels and social media platforms commonly used by program participants. This sampling method was deemed appropriate because it targets individuals with firsthand experience and relevant knowledge aligned with the objectives of the study, rather than aiming for random representation of the broader population. The total population of interest consisted of approximately 5,000 young individuals who had engaged with these innovation hub programs. Based on this population size, a sample of 357 respondents was selected for the study. The sample size was determined using the Krejcie and Morgan (1970) sample size determination table to estimate an appropriate sample size for the study population. Based on the sample size determination guidelines proposed by Krejcie and Morgan (1970), a minimum sample of 357 respondents was required for this study. The online survey gathered a total of 360 valid responses. However, the use of purposive non-probability sampling means that the findings should not be interpreted as statistically representative of all young adults in Somalia. Prior to analysis, the dataset was carefully reviewed to identify incomplete questionnaires or potential outliers; however, no significant missing values or anomalies were detected. The cleaned data were subsequently coded and transformed into numerical form to facilitate statistical analysis. All analyses were then performed using SPSS software (Table 1).

**Table 1:** Demographic information.

**Gender**	**Frequency**	**Percent**	**Age group**	**Frequency**	**Percent**
Male	194	53.9	20–25	169	46.9
Female	166	46.1	26–30	170	47.2
Total	360	100	31–35	21	5.8
			Total	360	100
**Education level**			**Current employment status**		
High school	26	7.2	Unemployed	4	1.1
Vocational school	10	2.8	Full-time employed	110	30.6
Bachelor degree	286	79.4	Part-time	34	9.4
Master degree	38	10.6	Freelancer	105	29.2
Total	360	100	Entrepreneur	107	29.7
			Total	360	100

### Data analysis

Data analysis for this study was conducted using Statistical Package for the Social Sciences (SPSS) version 29. Several statistical techniques were employed to address the research objectives and test the proposed hypotheses. These techniques included reliability analysis using Cronbach’s alpha, descriptive statistics to summarize respondent characteristics and key variables, Pearson correlation analysis to examine associations between variables, and multiple regression analysis to assess predictive relationships. Correlation analysis is widely used in empirical research to determine the strength and direction of relationships between variables. Linear correlation, commonly expressed through correlation coefficients (r or R), measures the degree to which two variables are related. Given its extensive application in social science research, this study focused primarily on linear correlation analysis. While correlation can take both linear and non-linear forms, the linear approach was deemed appropriate for examining the relationships among the study variables.

To further explore these relationships, the study employed multiple linear regression analysis, a standard analytical technique in social sciences that examines the effect of two or more independent variables on a single dependent variable. Skills Development and Training (SDT), Entrepreneurship Support (ES), and Freelancing Programs (FP) were treated as independent variables in this research, while Young Adults’ Employment (YAE) functioned as the dependent variable. This framework made it possible to examine the individual as well as the combined influence of innovation hub interventions on employment outcomes. In addition, the regression analysis included evaluations of model adequacy and robustness through measures such as the coefficient of determination (R^2^) and adjusted R^2^, tests of statistical significance using *p*-values, and diagnostics for multicollinearity using the Variance Inflation Factor (VIF) and tolerance values. These assessments were conducted to ensure the reliability and validity of the regression results. The multiple linear regression model applied in this study is expressed as follows:

### Multiple linear regression equation

Y: Dependent variable

β_0_: Intercept

X_1_, X_2_, X_3:_ Independent variables

*ε*: Error term

This study’s regression equation is as follows.


$$\mathrm{YAE}=\beta_{0}+\beta_{1}(\mathrm{SDT})+\beta_{2}(\mathrm{ES})+\beta_{3}(\mathrm{FP})+\varepsilon$$

## Results

### Pre-program situation result

The findings shown in Table 2 indicate that the majority of participants joined the innovation hub from a position of economic vulnerability. Over half of the respondents (54.7%) were unemployed before joining, while only a small proportion were in full-time employment (1.7%). A notable share were students (14.2%), part-time workers (18.3%), and freelancers (9.2%), suggesting that the hub primarily attracts individuals seeking to improve their labor market outcomes. Regarding job search duration, most participants had been seeking employment for extended periods: 38.3% reported being unemployed for more than one year, and 34.4% for 6–12 months. Only 7.2% had been unemployed for less than six months, indicating prolonged employment challenges among participants. The primary motivations for joining the hub were job-related and skill-oriented. Finding job opportunities was the most frequently cited reason (63.3%), followed by learning new skills (58.3%) and networking with peers (57.5%). Additionally, a substantial proportion (42.2%) joined with the intention of starting a business. Overall, the results provide descriptive evidence that many participants joined the innovation hub from economically vulnerable situations, highlighting the potential relevance of innovation hub programs in supporting skills development, entrepreneurship, and employment-related opportunities. However, these descriptive findings should not be interpreted as direct evidence of employment improvement, as no formal before-and-after comparison was conducted.

**Table 2:** Pre-program situation result.

**Employment status before joining the innovation hub**	**Frequency**	**%**	**Employment seeking period before joining the hub**	**Frequency**	**%**
Unemployed	197	54.7	Less than 6 months	26	7.2
Full-time employed	6	1.7	6–12 Months	124	34.4
Part-time	66	18.3	More than 1 year	138	38.3
Freelancer	33	9.2	Not looking for jobs at all	72	20.0
Entrepreneur	7	1.9	Total	360	100
Student	51	14.2			
Total	360	100			
**Reasons for joining** **the hub**					
To find job opportunities	228	63.3			
To learn new skills	210	58.3			
To network with other peers	201	57.5			
To start a business	152	42.2			

### Program participation insights

The distribution of participants across innovation hubs was relatively balanced, as shown in Table 3, with 51.7% attending SIMAD iLab and 48.3% attending iRise Hub. In terms of participation duration, most respondents had substantial engagement with the hubs, as 54.7% reported participation lasting 8–12 months and 39.7% between 4 and 8 months. Only a small proportion participated for less than four months (1.4%) or more than one year (4.2%). This suggests that the majority of participants remained engaged with the hubs for a medium to long-term period.

**Table 3:** Program participation insights.

**Innovation hubs**	**Frequency**	**%**	**Participation period**	**Frequency**	**%**
SIMAD iLab	186	51.7	2–4 Months	5	1.4
iRise hub	174	48.3	4–8 Months	143	39.7
Total	360	100	8–12 Months	197	54.7
			More than 1 year	15	4.2
			Total	360	100.0

### Descriptive statistics

Descriptive statistics for the study variables are displayed in Table 4, with all items measured on a five-point Likert scale from 1 (strongly disagree) to 5 (strongly agree). Skills Development and Training (SDT) recorded a mean of 4.25 and a standard deviation of 0.46, reflecting strong agreement among participants regarding the value of the skills training delivered by innovation hubs. The relatively low standard deviation suggests a strong level of consistency in participants’ perceptions. Similarly, Entrepreneurship Support (ES) recorded a mean value of 4.26 and a standard deviation of 0.48, reflecting both strong and consistent agreement about the effectiveness of entrepreneurial support services offered. Freelancing Programs (FP) yielded a slightly lower mean score of 4.18, accompanied by a standard deviation of 0.46, indicating generally positive but marginally less strong perceptions compared to the other services. The dependent variable, Young Adults’ Employment (YAE), recorded a mean score of 4.20 with a standard deviation of 0.38, the lowest variability among the variables, suggesting that most participants perceived an improvement in their employment outcomes as a result of participating in innovation hub programs.

**Table 4:** Descriptive statistics insights.

Variables	*N*	Mean	Std. deviation
SDT	360	4.2514	0.45602
ES	360	4.2562	0.48458
FP	360	4.1819	0.45732
YAE	360	4.2000	0.38060

The dependent variable, Young Adults’ Employment (YAE), was operationalized as participants’ perceived employment-related outcomes following participation in innovation hub programs. The construct was measured using three questionnaire items assessing respondents’ self-reported employment attainment, income stability, and employment transition experiences after engaging in innovation hub activities. Specifically, the items measured whether participants perceived themselves as currently employed; whether they had achieved stable or consistent income through employment, freelancing, or business activities; and whether they had transitioned from unemployment to employment or self-employment following program participation. Responses were measured using a five-point Likert scale ranging from 1 = Strongly Disagree to 5 = Strongly Agree. Composite scores were computed by averaging the item responses, allowing the construct to be treated as a continuous variable for correlation and multiple linear regression analyses. Accordingly, the YAE construct reflects perceived employment-related outcomes rather than direct objective measures of long-term employment quality or labor-market stability.

### Reliability analysis

The reliability of all study variables, evaluated through Cronbach’s alpha, is summarized in Table 5. The results reveal strong internal consistency for all independent variables. Skills Development and Training attained an alpha coefficient of 0.806, signifying high reliability across the four items comprising this construct. Entrepreneurship Support similarly demonstrated strong reliability with an alpha of 0.809. Freelancing Programs recorded a coefficient of 0.774, which remains within the accepted range for reliable internal consistency. Collectively, these results suggest that the measurement items for the three independent constructs are well correlated and effectively capture the intended concepts.

**Table 5:** Reliability analysis.

Variables	Cronbach’s Alpha	Cronbach’s Alpha based on standardized items	N of items
SDT	0.806	0.807	4
ES	0.809	0.809	4
FP	0.774	0.774	4
YAE	0.642	0.643	3

The dependent variable, Young Adults’ Employment (YAE), recorded a Cronbach’s alpha of 0.642. Although this value is slightly below the conventional threshold of 0.70, it remains acceptable for exploratory research. According to Hair et al. (2010) reliability coefficients between 0.60 and 0.70 are considered acceptable in exploratory studies, particularly in social science research where constructs are being examined in relatively underexplored contexts. Similarly, Nunnally and Bernstein (1994) note that lower reliability thresholds may be acceptable in the early stages of theory development and exploratory investigation. Furthermore, Cronbach’s alpha is sensitive to the number of items in a scale, and shorter scales often produce lower alpha coefficients despite acceptable internal consistency (Cortina, 1993). Since the YAE construct in this study was measured using only three items, the relatively lower alpha coefficient is not unexpected. Given the exploratory nature of this study and the limited prior empirical work on innovation hubs and employment outcomes in Somalia, the reliability level of the YAE construct was considered sufficient for analysis.

To assess the potential presence of common method bias resulting from the use of self-reported questionnaire data collected from a single source, Harman’s single-factor test was conducted using all measurement items included in the study. As presented in Table 6, the unrotated principal component analysis revealed that the first factor accounted for 39.696% of the total variance, which is below the commonly accepted threshold of 50%. This suggests that common method bias was unlikely to pose a serious threat to the validity of the study findings.

**Table 6:** Harman’s single-factor test results.

Component	Eigenvalue	Variance explained (%)	Cumulative variance (%)
1	1.588	39.696	39.696
2	1.001	25.015	64.711

Extraction method: principal component analysis.

### Assumption testing

Prior to conducting the multiple linear regression analysis, several diagnostic procedures were performed to assess whether the assumptions of multiple linear regression were reasonably satisfied. The assumptions of normality, linearity, homoscedasticity, and independence of errors were examined using residual plots, histograms, normal probability plots, and the Durbin-Watson statistic.

**Figure 2:**
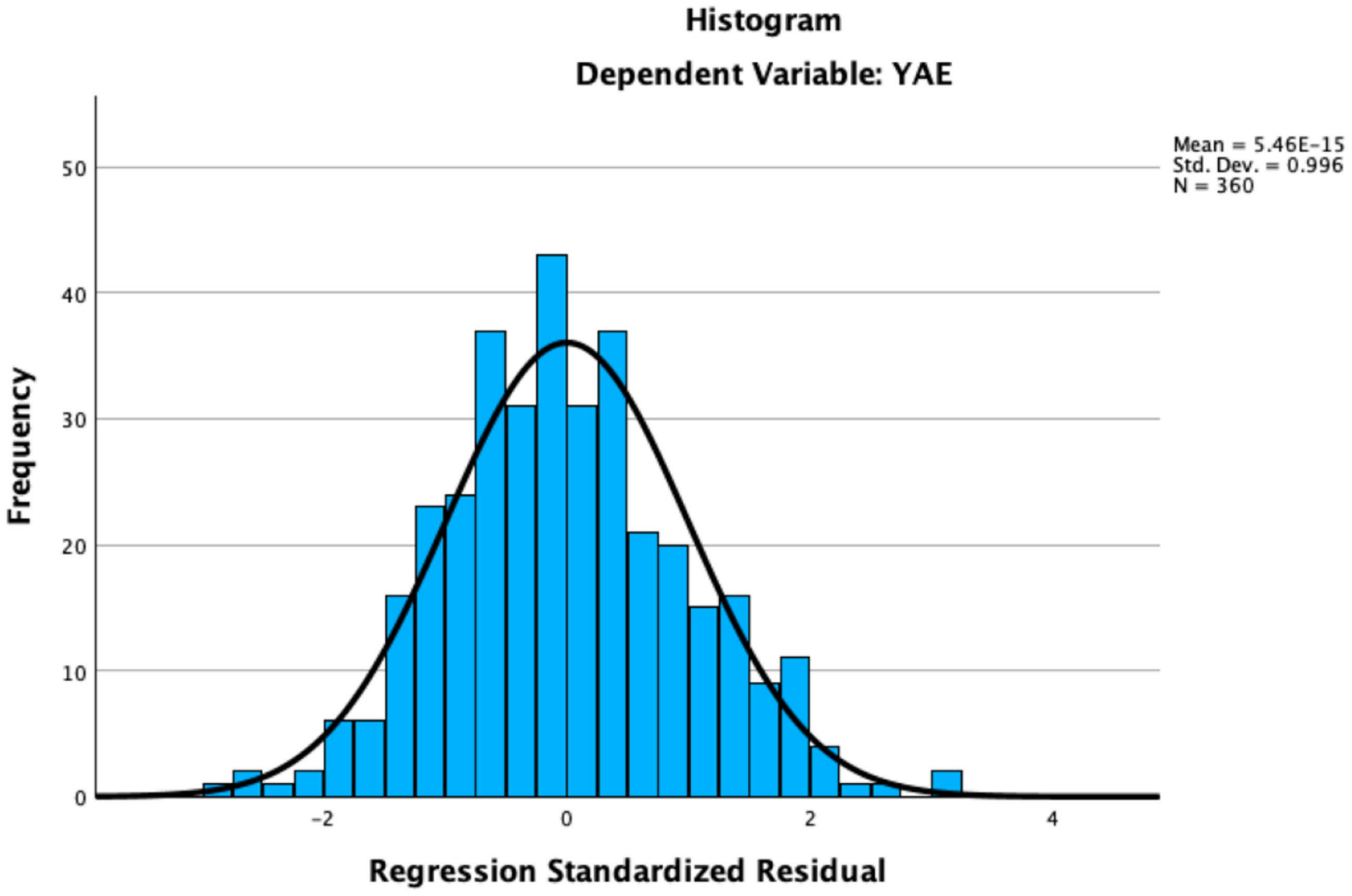
Histogram of standardized residuals.

In addition, multicollinearity was reassessed using tolerance and Variance Inflation Factor (VIF) values. The diagnostic results indicated no serious violations of the regression assumptions, suggesting that the data were suitable for multiple linear regression analysis (Figure 2).

**Figure 3:**
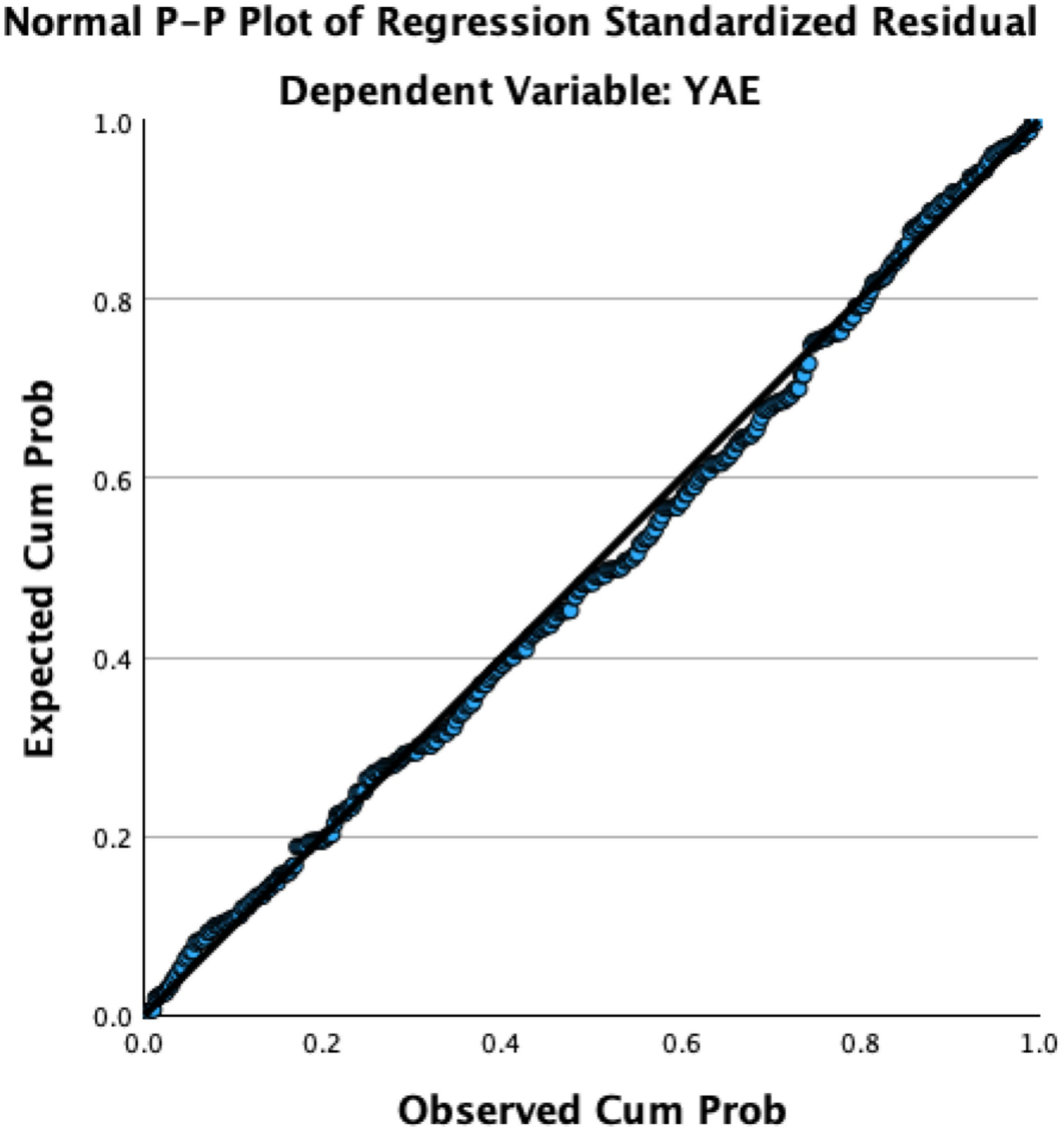
Normal P–P plot of regression standardized residuals.

The histogram of standardized residuals demonstrated an approximately normal distribution, supporting the normality assumption of multiple regression (Figure 3).

**Figure 4:**
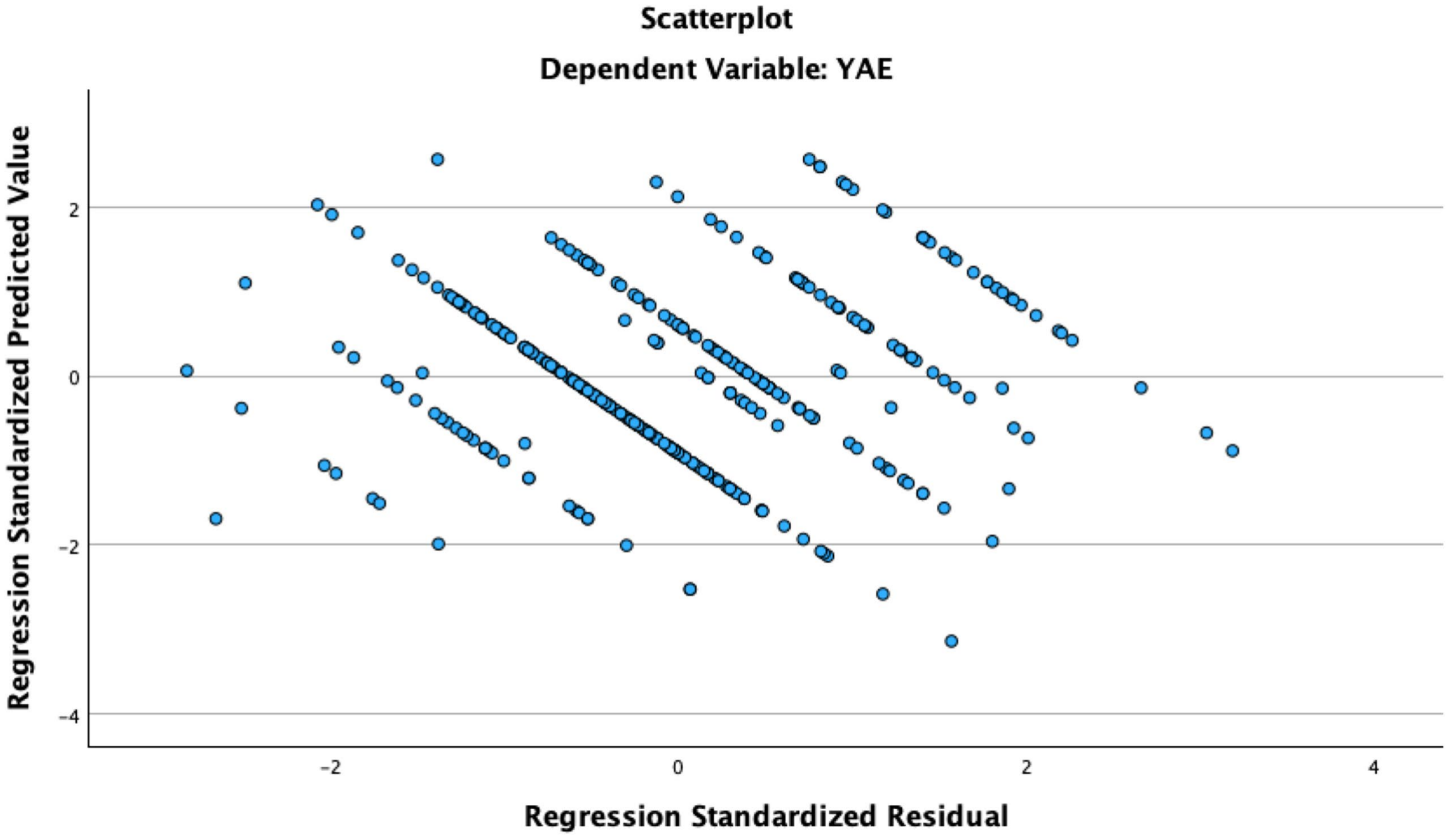
Scatterplot of standardized residuals.

The normal P–P plot indicated that the residual values closely followed the diagonal line, suggesting that the residuals were approximately normally distributed (Figure 4).

The scatterplot of standardized residuals showed no obvious funnel shape or systematic pattern, indicating that the assumptions of linearity and homoscedasticity were reasonably satisfied.

Furthermore, the Durbin-Watson value of 2.119 indicated no evidence of serious autocorrelation in the residuals, suggesting that the independence of errors assumption was reasonably satisfied.

### Correlation analysis

The Pearson correlation analysis results, presented in Table 7, were used to assess the direction and strength of linear relationships between the independent and dependent variables. Pearson’s correlation coefficient is well-suited for evaluating relationships among continuous variables, such as the composite Likert-scale measures employed in this study. The findings reveal positive and statistically significant relationships between each of the independent variables.

**Table 7:** Correlation analysis.

**Variables**		**SDT**	**ES**	**FP**	**YAE**
**SDT**	Pearson Correlation	1	0.010	0.000	0.376**
	Sig. (2-tailed)		0.846	0.994	<0.001
	N	360	360	360	360
**ES**	Pearson Correlation	0.010	1	0.011	0.308**
	Sig. (2-tailed)	0.846		0.830	<0.001
	N	360	360	360	360
**FP**	Pearson Correlation	0.000	0.011	1	0.319**
	Sig. (2-tailed)	0.994	0.830		<0.001
	N	360	360	360	360
**YAE**	Pearson Correlation	0.376**	0.308**	0.319**	1
	Sig. (2-tailed)	<0.001	<0.001	<0.001	
	N	360	360	360	360

**indicates that the correlation is significant at the 0.01 level (2-tailed).

Specifically, SDT exhibited a moderate positive correlation with YAE (r = 0.376), which was statistically significant at the 0.01 level (*p* < 0.001). Similarly, ES showed a positive association with YAE, with a correlation coefficient of r = 0.308, also significant at the 0.01 level (*p* < 0.001), although the strength of this relationship was slightly weaker than that observed for SDT. In addition, FP demonstrated a positive and statistically significant correlation with YAE (r = 0.319, *p* < 0.001), indicating that freelancing-related interventions are positively associated with improved employment outcomes among young adults.

Importantly, the correlations among the independent variables themselves were negligible and statistically insignificant (SDT–ES: r = 0.010, *p* = 0.846; SDT–FP: r = 0.000, *p* = 0.994; ES–FP: r = 0.011, *p* = 0.830). These low intercorrelations suggest that the independent variables operate largely independently of one another. Such a pattern is desirable in regression analysis, as it reduces the likelihood of multicollinearity and strengthens the reliability of the regression estimates.

### Regression analysis

#### Model summary

Table 8 presents the model summary from the multiple linear regression analysis conducted to assess the combined influence of the three independent variables on the dependent variable. While the table contains several statistical indicators, this discussion focuses on the coefficient of determination (R^2^) and the adjusted R^2^, as these measures are most relevant for evaluating the explanatory strength of the model.

- The R-squared value (R^2^ = 0.333) indicates that approximately 33.3% of the variation in YAE can be explained by the combined effects of SDT, ES, and FP. Although this level of explained variance is moderate, it is generally considered acceptable within the context of social science research. Cohen (2013) suggests that R-squared values of 0.26 or higher represent medium to large effect sizes in behavioral studies. Similarly, Ozili (2022) argues that R-squared values ranging from 0.10 to 0.50 are appropriate for social science models, particularly when key explanatory variables demonstrate statistical significance.
- The adjusted R-squared value (Adjusted R^2^ = 0.328) is slightly lower than the R-squared value, as it accounts for both the number of predictors included in the model and the sample size. The minimal difference between R^2^ and adjusted R^2^ indicates that the model does not suffer from overfitting, suggesting that it provides a reliable and well-specified explanation of the relationship between the predictors and the outcome variable.
- In addition, the Durbin-Watson value of 2.119 indicates no evidence of serious autocorrelation in the residuals, suggesting that the independence of errors assumption was reasonably satisfied.

**Table 8:** Model summary.

Model	R	R square	Adjusted R square	Std. error of the estimate	Durbin-Watson
1	0.577^a^	0.333	0.328	0.31209	2.119

^a^Dependent variable: Young adults’ employment (YAE).

#### ANOVA table

Table 9 reports the Analysis of Variance (ANOVA) results for the multiple linear regression model used to assess the overall significance of predicting Young Adults’ Employment based on three independent variables. Although the full ANOVA output is presented in the table, the discussion focuses specifically on the F-statistic and its corresponding significance level, as these indicators are central to evaluating the model’s overall explanatory validity. The regression model produced an *F*-value of 59.303, indicating strong explanatory power and demonstrating that the combined set of independent variables significantly contributes to predicting YAE. A high F-statistic reflects a good overall model fit and suggests that the regression equation meaningfully explains variation in the dependent variable. In addition, the associated significance level (*p* < 0.001) confirms that the model is statistically significant. This result indicates that the probability of observing such a relationship between the predictors and young adults’ employment by chance alone is less than 0.1%. Consequently, the findings provide strong evidence that SDT, ES, and FP collectively have a significant effect on employment outcomes among young adults.

**Table 9:** ANOVA table^a^.

Model	Sum of squares	df	Mean square	F	Sig.
Regression	17.328	3	5.776	59.303	<.001^b^
Residual	34.674	356	0.097		
Total	52.003	359			

^a^Dependent variable: YAE.

^b^Predictors: FP, SDT, ES.

#### Coefficient table

Table 10 presents the regression coefficients for the multiple linear regression model, indicating that all three independent variables exert a statistically significant and positive influence on the dependent variable. This conclusion is supported by the significance values (*p*-values) for independent variables, all of which are below 0.001, confirming their statistical significance. These results demonstrate that each independent variable makes a meaningful contribution to the explanatory power of the model.

**Table 10:** Coefficient table^a^.

Model	Unstandardized coefficients		Standardized coefficients			Collinearity statistics	
	B	Std. error	Beta	t	Sig.	Tolerance	VIF
(Constant)	0.768	0.258		2.974	0.003		
SDT	0.312	0.036	0.373	8.608	<0.001	1.000	1.000
ES	0.236	0.034	0.300	6.942	<0.001	1.000	1.000
FP	0.263	0.036	0.316	7.298	<0.001	1.000	1.000

^a^Dependent variable: YAE.

The unstandardized regression coefficients (B) show that a one-unit increase in Skills Development and Training is associated with a 0.312-unit increase in Young Adults’ Employment (YAE), holding all other variables constant. Similarly, Entrepreneurship Support has a B coefficient of 0.236, while Freelancing Programs exhibit a B value of 0.263, indicating positive effects on employment outcomes. The standardized coefficients (Beta) further illustrate the relative influence of each predictor. Skills Development and Training has the strongest standardized effect (*β* = 0.373), followed by Freelancing Programs (*β* = 0.316) and Entrepreneurship Support (*β* = 0.300). The positive beta coefficients indicate that increases in each independent variable correspond to improvements in YAE.

Collinearity diagnostics were also examined to assess the stability of the regression estimates. The tolerance values for all three predictors were 1.000, and the corresponding Variance Inflation Factor (VIF) values were also 1.000. These results indicate the absence of multicollinearity among the independent variables, confirming that each predictor independently contributes to the model without inflating standard errors or distorting coefficient estimates.

## Discussion

The regression results indicate that Skills Development and Training (SDT) is significant and positively associated with young adults’ employment outcomes (see Table 10). The unstandardized coefficient (B = 0.312) implies that a one-unit increase in SDT is associated with a corresponding increase of 0.312 units in a young adult’s employment, holding other variables constant. The standardized beta coefficient (*β* = 0.373) reflects the relative contribution of SDT to explaining variation in employment outcomes when considered alongside the other predictors in the model. In addition, the t-value (t = 8.608) and the associated *p*-value (*p* < 0.001) confirm that this relationship is highly statistically significant. Accordingly, Hypothesis 1 is supported.

The findings suggest that young adults with higher levels of skills acquisition, particularly in areas such as graphic design, mobile application development, and web development, tend to exhibit greater competitiveness in the labor market. In this context, training provided by innovation hubs appears to complement traditional academic education in equipping young adults with the practical capabilities that employers demand. The results reinforce earlier empirical evidence within the Somali context and highlight skills-based training as a viable strategy for addressing youth unemployment. The findings suggest that innovation hubs may complement formal academic education by providing practical, market-oriented skills, entrepreneurship exposure, and digital work opportunities that are not always sufficiently emphasized within traditional educational settings.

Moreover, these outcomes strongly align with the principles of human capital theory (Becker, 1962), which posits that investments in education and training enhance individual productivity and employment prospects. From this perspective, innovation hub programs represent direct investments in human capital that have positive labor market returns. The findings support the theoretical proposition that employability is closely linked to the accumulation of relevant skills and competencies. Consequently, skills development and training initiatives offered by innovation hubs emerge as innovative and effective mechanisms for improving a young adult’s employment in Somalia, while significantly strengthening young adults’ employability through flexible and context-appropriate training models. The findings, therefore, provide empirical support for the central proposition of human capital theory that investments in knowledge, practical skills, and training enhance individuals’ productive capabilities and improve their labor market opportunities, particularly in fragile and resource-constrained contexts.

The regression analysis further reveals that Entrepreneurship Support (ES) has a statistically significant and positive effect on young adults’ employment (see Table 10). The unstandardized coefficient (B = 0.236) indicates that a one-unit increase in entrepreneurship support leads to a 0.236-unit increase in youth employment outcomes. The standardized beta coefficient (*β* = 0.300) suggests a moderate effect size, while the t-value (t = 6.942) and *p*-value (*p* < 0.001) confirm strong statistical significance. These results provide empirical support for Hypothesis 2.

The findings demonstrate that entrepreneurship support services offered through innovation hubs play a critical role in enhancing a young adult’s employment, particularly by lowering entry barriers to business creation in developing economies such as Somalia. Through structured entrepreneurship programs, young people gain essential knowledge, confidence, mentoring, and access to professional networks that enable them to initiate and sustain business ventures. In contexts where formal employment opportunities are limited, entrepreneurship presents a viable and sustainable pathway for youth economic participation. The results of this study are consistent with prior research highlighting the positive role of entrepreneurial support in employment creation. For instance, Malenya et al. (2025) emphasize the importance of incubators that provide mentorship, access to finance, and business development services in facilitating youth-led enterprise creation.

These findings also align with the principles of Effectuation Theory, which emphasizes entrepreneurial action under conditions of uncertainty through the utilization of available resources, networks, and experiential learning. The entrepreneurship support initiatives offered by innovation hubs, including mentorship, business guidance, networking opportunities, and startup-oriented training, may assist young adults in developing adaptive entrepreneurial competencies within Somalia’s fragile and resource-constrained economic environment. From this perspective, the findings suggest that innovation hubs may contribute to strengthening entrepreneurial capabilities by supporting opportunity recognition, practical experimentation, and resource mobilization among young adults.

These findings further demonstrate how innovation hubs create enabling environments in which young adults can utilize existing resources, networks, mentorship, and incremental experimentation to pursue entrepreneurial opportunities despite uncertainty and institutional constraints, reflecting the core principles of Effectuation Theory within the Somali context.

Similarly, the results indicate that Freelancing Programs are statistically significantly and positively associated with young adults’ employment outcomes (see Table 10). The unstandardized coefficient (B = 0.263) suggests that a one-unit increase in participation in freelancing programs leads to a 0.263-unit improvement in employment outcomes. The standardized beta coefficient (*β* = 0.316) reflects a moderate-to-strong effect, while the t-value (t = 7.298) and p-value (*p* < 0.001) confirm the robustness of this relationship.

Accordingly, Hypothesis 3 is supported. These findings underscore the increasing relevance of the gig economy in fragile and developing labor markets such as Somalia, where access to stable, full-time employment remains limited. Freelancing initiatives run by innovation hubs provide young individuals with remote-working capabilities, allowing them to tap into flexible earning opportunities via digital platforms such as Upwork and Fiverr. The gig economy offers a practical entry point into paid work for many young people, especially those living in economically disadvantaged environments (Pesole et al., 2018). New entrants to online freelancing, often referred to as “newbies” or “noobs,” typically begin by treating digital work as a part-time or secondary income source while continuing to explore local job opportunities (Sison and Lavilles, 2018). The findings further correspond with the assumptions of Gig Economy Theory, which highlights the growing role of flexible digital work arrangements and online labor market participation in shaping contemporary employment opportunities. In the Somali context, freelancing programs provided through innovation hubs may assist young adults in accessing global digital work opportunities despite local labor-market constraints and limited formal employment availability. The findings, therefore, suggest that freelancing initiatives may expand alternative income-generating pathways for young adults within fragile and digitally evolving economic environments.

Overall, the findings suggest that innovation hubs may play an important role in supporting employment-related outcomes among young adults participating in innovation hub programs in Mogadishu, Somalia.

While the findings suggest positive associations between innovation hub participation and employment-related outcomes, the present study did not directly examine the long-term quality, stability, or sustainability of employment obtained through freelancing, entrepreneurship, or other forms of work. In fragile and digitally evolving labor-market environments, freelancing and platform-based work may provide important income-generating opportunities while also exposing young adults to challenges such as income instability, irregular work availability, limited labor protections, and global market competition. Therefore, the findings should be interpreted cautiously, as employment attainment does not necessarily guarantee decent work conditions or long-term economic security.

### The study’s contribution to the literature

This study makes a substantial contribution to the literature on young adults’ employment by examining the role of innovation hub programs in Somalia. While earlier research has acknowledged the influence of skills development, entrepreneurship, and freelancing initiatives on employment outcomes, there remains a scarcity of predictive, empirically grounded studies focusing on these interventions within the Somali context. By concentrating specifically on innovation hubs such as SIMAD iLab and iRise Hub, this research addresses an important gap by providing evidence on the employment-creation capacity of innovation-driven institutions in developing and post-conflict economies.

The findings reinforce the importance of practical skills training and entrepreneurship support as critical pathways to employment, consistent with prior studies by Özocaklı et al. (2025), Zaman (2015), Medun and Bello (2024), Peeters (2009), Youmans et al. (2024). Building on this body of work, the present study extends the literature by demonstrating how innovation hubs operationalize these interventions through structured programs that directly translate into employment opportunities for young people, an area that remains underexplored in existing research. This contribution is particularly relevant in Somalia, where formal employment systems are limited and young adults increasingly rely on digital, remote, and flexible forms of work to secure livelihoods.

In addition, the study advances theoretical understanding by integrating and empirically validating three complementary frameworks, Human Capital Theory, Effectuation Theory, and Gig Economy Theory, within a single analytical model. By doing so, it demonstrates how these theories collectively explain a young adult’s employment dynamics in a fragile, low-income context and illustrates how theoretical concepts can be translated into practical interventions within innovation hub environments. The empirical evidence generated also provides a foundation for comparative studies in other post-conflict and developing countries facing similar labor market challenges.

Ultimately, this research enriches the literature on integrated approaches to a young adult’s employment by highlighting the synergistic effects that emerge when skills development, entrepreneurship support, and freelancing opportunities are implemented together. Rather than viewing employability through a narrow, linear job-placement lens, the study supports a more holistic understanding of youth employment, one that recognizes the interconnected nature of skills, entrepreneurial capacity, and digital work in shaping sustainable employment outcomes.

## Conclusions and recommendations

### Conclusion

This study examined the relationship between innovation hub programs and young adults’ employment outcomes in Somalia, focusing specifically on programs delivered by SIMAD iLab and iRise Hub. A quantitative methodology was adopted, employing a structured online survey of data from young adults who had taken part in programs offered by the innovation hubs, analyzed with SPSS. The findings demonstrate that all three innovation hub programs have statistically significant and positive effects on a young adult’s employment outcomes. Skills training had the strongest effect, followed by freelancing and entrepreneurship support. Taken together, the findings provide empirical evidence regarding the potential role of innovation hubs in supporting youth employment and economic participation among young adults in Mogadishu, Somalia.

### Recommendations

The recommendations outlined below are intended to strengthen the role of hubs in addressing young adults’ unemployment in Somalia.

### Policy and practical recommendations

- Innovation hubs should expand practical and market-oriented skills training programs that align with current labor market demands and equip young adults with relevant employability competencies.
- Innovation hubs and relevant stakeholders should strengthen support for freelancing and digital work opportunities by enhancing digital skills training and improving young adults’ access to global online labor markets.
- Innovation hubs should strengthen entrepreneurship support initiatives through mentorship, startup incubation, networking opportunities, and access to business development resources for young adults.

Collectively, these recommendations aim to reposition innovation hubs as strategic instruments for addressing youth unemployment in Somalia, extending their role beyond local initiatives to become integral components of national employment solutions. By implementing these measures, stakeholders can contribute to creating a more inclusive, resilient, and opportunity-driven environment for Somali youth. Furthermore, this study offers both strategic and practical insights for policymakers and practitioners, while also providing a valuable foundation for future research on employment generation in developing and post-conflict contexts.

### Limitations of the study

Despite its contributions, this study has several limitations that warrant consideration. First, the research focused exclusively on the hubs located in Mogadishu. Although these hubs are among the most prominent in the city, the findings may not be fully generalizable to innovation hubs operating in other regions of Somalia, where contextual conditions and program structures may differ. Second, the limitation relates to the use of purposive sampling as the participant selection strategy. Although this approach was appropriate for targeting respondents with direct experience in innovation hub programs, it may introduce selection bias and limit the generalizability of the findings beyond the sampled population. Since participants were intentionally selected based on their engagement with innovation hubs, the results may not fully represent the broader population of young adults in Somalia, particularly those who have not participated in such programs. Third, the study relied on self-reported data collected through an online questionnaire using a cross-sectional research design. Such an approach may be subject to recall bias, as respondents could overstate the perceived benefits of the programs they participated in or provide responses aligned with expected positive outcomes, potentially influencing the accuracy of the results. In addition, the cross-sectional nature of the study limits the ability to assess whether the employment outcomes reported by participants are sustained over time. Since data were collected at a single point in time, the study cannot determine the long-term stability, continuity, or progression of employment after participation in innovation hub programs. Future longitudinal studies are recommended to track employment outcomes over extended periods and provide deeper insights into the sustainability of these outcomes.

Furthermore, although the study collected descriptive information on participants’ employment situations prior to joining innovation hubs, it did not employ a formal before-and-after comparative design. As a result, changes in employment outcomes over time cannot be directly attributed or statistically compared within this study.

Another important limitation of this study is the absence of a control or comparison group involving young adults who did not participate in innovation hub programs. As a result, the study cannot conclusively determine whether the observed employment-related outcomes are directly attributable to innovation hub participation or influenced by other external factors such as prior education, personal motivation, social networks, labor-market conditions, or previous work experience. Consequently, the findings should be interpreted as associative rather than causal. Future studies are encouraged to incorporate comparative or longitudinal research designs to provide stronger evidence regarding employment-related outcomes associated with innovation hub participation. Another limitation of this study is that the regression model focused primarily on innovation hub service variables and did not incorporate additional control variables such as age, prior work experience, socioeconomic background, or other contextual factors that may also influence employment outcomes. Although the selected variables were aligned with the study objectives, the exclusion of potential control variables may limit the comprehensiveness of the model. Future studies are encouraged to incorporate broader demographic and contextual controls to provide a more comprehensive understanding of the factors influencing young adults’ employment. As such, the exclusion of these variables may increase the possibility of omitted-variable bias within the regression estimates.

Finally, the study employed a purely quantitative research approach and did not incorporate qualitative methods, such as in-depth interviews or focus group discussions. As a result, the analysis may not fully capture participants’ lived experiences, including perceptions of training quality, challenges encountered in freelancing or entrepreneurship, and contextual factors influencing program effectiveness. Integrating qualitative insights could have enriched the interpretation of the findings.

### Future research suggestions

Building on the findings of this study, several avenues for future research are recommended to deepen the understanding of the role of innovation hubs in a young adult’s employment in Somalia. First, future studies should adopt mixed-methods approaches that combine quantitative surveys with qualitative techniques such as interviews, focus group discussions, and case studies. This would allow for a more comprehensive exploration of youth experiences and provide richer contextual insights. Second, future research should broaden the geographical scope beyond Mogadishu to include other cities and regions where innovation hubs are emerging. Examining regional variations would help identify contextual factors influencing program effectiveness and enhance the generalizability of findings. Finally, comparative studies are encouraged that include both participants who have benefited from innovation hub programs and young adults who have not engaged with such initiatives. Such comparisons would provide stronger evidence of causal relationships and offer more robust insights into the true impact of innovation hubs on a young adult’s employment outcomes.

## Data Availability

The raw data supporting the conclusions of this article will be made available by the authors, without undue reservation.
